# (1*R*,4*R*,5a*S*,7*S*,9a*S*)-7,9a-Dimethyl-6-methyl­ene-3-oxo-1,3,4,5,5a,6,7,8,9,9a-deca­hydro­naphtho­[1,2-*c*]furan-1,4-diyl diacetate

**DOI:** 10.1107/S1600536812033636

**Published:** 2012-08-01

**Authors:** Mercy Mudyiwa, Mohamed S. Rajab, Frank R. Fronczek, Steven F. Watkins

**Affiliations:** a Department of Chemistry, Louisiana State University, Baton Rouge, LA 70803-1804, USA

## Abstract

The title compound, C_19_H_24_O_6_, is a sesquiterpene lactone isolated from the Kenyan plant *Warburgia ugandensis*. Ring *A* adopts a chair conformation, ring *B* is in a *C*_2_ twist conformation and the lactone ring is nearly planar with maximum deviation 0.007 (1) Å. The reported absolute configuration is based on that of the similar compound bromo-parasiticolide A and is supported by analysis of Bijvoet differences from light atoms in Mo *K*α radiation.

## Related literature

For related structures, see: Fukuyama *et al.* (1975 ▶) (Bromo-parasiticolide A; PARASB); Ikhiri *et al.* (1995 ▶) (ZOXLIH); Aranda *et al.* (2001 ▶) (ABUKIR); King *et al.* (1973 ▶) (PRPRDE); Rossmann & Lipscomb (1958 ▶) (IRSBBZ); Rahbaek *et al.* (1997 ▶) (NEYKOR), Zhang *et al.* (2006 ▶) (UCOLAA, UCOKUT); Harinantenaina *et al.* (2007 ▶) (NIDJUG); McCorkindale *et al.* (1981 ▶) (PEBRLD); Hayashi *et al.* (2010 ▶) (VUTCIX). For the absolute configuration of sesquiterpene lactones, see: Fischer *et al.* (1979 ▶). For a description of the Cambridge Structural Database, see: Allen (2002 ▶). For the absolute configuration from Bijvoet pairs, see: Hooft *et al.* (2008 ▶). For compounds from *Warburgia ugandensis*, see: Wube *et al.* (2005 ▶) and for related compounds, see: Garland (1969 ▶); Kokwaro (1976 ▶).
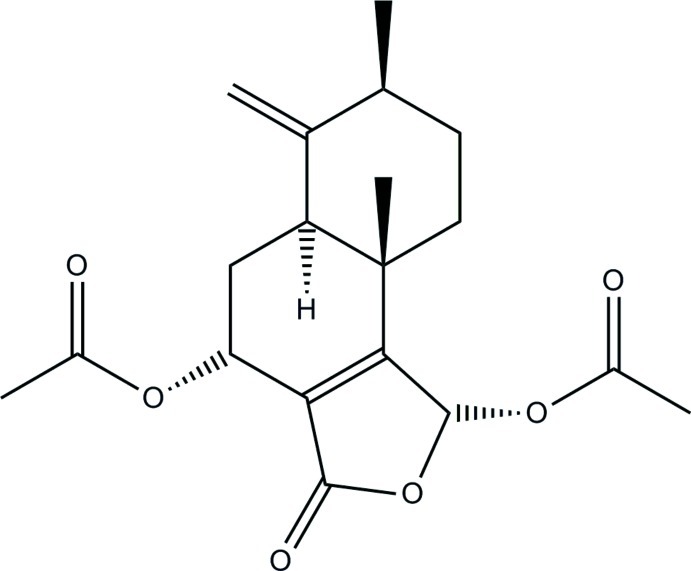


## Experimental

### 

#### Crystal data

C_19_H_24_O_6_
*M* *_r_* = 348.38
Tetragonal, 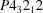

*a* = 13.014 (2) Å
*c* = 21.167 (3) Å
*V* = 3584.9 (9) Å^3^
*Z* = 8
Mo *K*α radiation
μ = 0.10 mm^−1^
*T* = 100 K
0.37 × 0.25 × 0.25 mm

#### Data collection

Nonius KappaCCD diffractometer
Absorption correction: multi-scan (*SCALEPACK*; Otwinowski & Minor, 1997 ▶) *T*_min_ = 0.966, *T*_max_ = 0.977
11378 measured reflections
6507 independent reflections
5934 reflections with *I* > 2σ(*I*)
*R*_int_ = 0.021

#### Refinement

*R*[*F* ^2^ > 2σ(*F* ^2^)] = 0.036
*wR*(*F* ^2^) = 0.092
*S* = 1.02
6507 reflections
231 parameters
H-atom parameters constrained
Δρ_max_ = 0.28 e Å^−3^
Δρ_min_ = −0.21 e Å^−3^
Absolute structure: Flack (1983 ▶). 2776 Bijvoet pairs
Flack parameter: 0.4 (6)

### 

Data collection: *COLLECT* (Nonius, 2000 ▶); cell refinement: *DENZO* and *SCALEPACK* (Otwinowski & Minor, 1997 ▶); data reduction: *DENZO* and *SCALEPACK*; program(s) used to solve structure: *SIR2002* (Burla *et al.*, 2003 ▶); program(s) used to refine structure: *SHELXL97* (Sheldrick, 2008 ▶); molecular graphics: *ORTEP-3 for Windows* (Farrugia, 1997 ▶); software used to prepare material for publication: *WinGX* (Farrugia, 1999 ▶).

## Supplementary Material

Crystal structure: contains datablock(s) global, I. DOI: 10.1107/S1600536812033636/bt5979sup1.cif

Structure factors: contains datablock(s) I. DOI: 10.1107/S1600536812033636/bt5979Isup2.hkl

Supplementary material file. DOI: 10.1107/S1600536812033636/bt5979Isup3.cml

Additional supplementary materials:  crystallographic information; 3D view; checkCIF report

## Figures and Tables

**Fig. 1.**
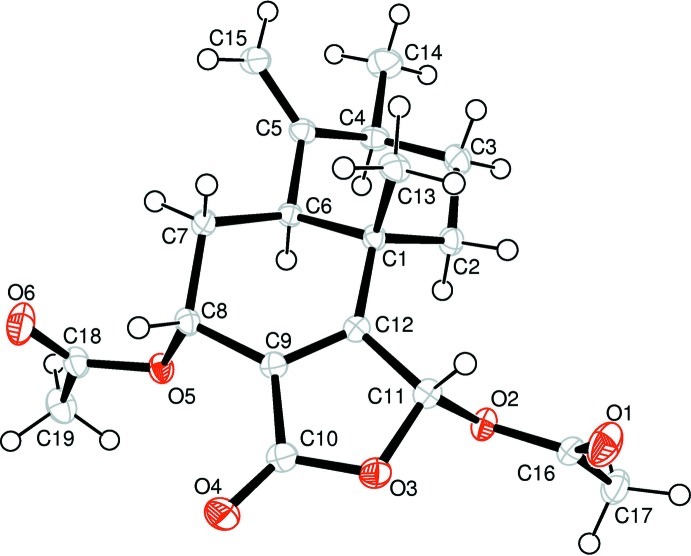
View of (I) (50% probability displacement ellipsoids)

## supplementary crystallographic information

### Comment

Coloratanolide and drimanolide sesquiterpene lactones, such as title compound I, have been isolated from the stem bark of *Warburgia ugandensis Sprague* (*Canellaceae*) as described by Wube *et al.* (2005). Plants of the genus Warburgia are of interest because of their use by herbalists in Kenya for the treatment of a number of parasitic diseases (Kokwaro, 1976). Compound I is the first sesquiterpene lactone to be crystallographically characterized which has the coloratanolide skeleton, (CAS 60306–54-9). The absolute configuration reported herein is based on the configuration of bromo-parasiticolide A (Fukuyama *et al.*, 1975), CCDC refcode PARASB (Allen, 2002), and supported by analysis of 2776 Bijvoet pairs.

### Experimental

Compound I was isolated from the stem bark of *Warburgia ugandensis Sprague* (*Canellaceae*) collected in Eldoret, Uasin Gishu District, Kenya. Crystals suitable for diffraction were grown from acetone/hexane/ethyl ether.

### Refinement

H atoms were placed in calculated positions, guided by difference maps, with C—H bond distances 0.95–1.00 Å, *U*_iso_ = 1.2*U*_eq_ of the attached carbon atom (1.5 for methyl), and thereafter treated as riding. A torsional parameter was refined for each methyl group.

The absolute configuration and space group assignment were established in part by analysis of 2776 Bijvoet pairs. Although the refined Flack parameter *x* = 0.4 (6) (Flack, 1983) is not definitive, the Hooft parameter *y* = 0.1 (3) and Hooft P2(true) = 0.998 (Hooft *et al.*, 2008) are strong indicators that the reported configuration is correct. This configuration is consistent with that of bromo-parasiticolide A (Fukuyama *et al.*, 1975), CCDC refcode PARASB (Allen, 2002) and with the accepted configuration of sesquiterpene lactones from higher plants (Fischer *et al.*, 1979).

### Crystal data

**Table:**

C_19_H_24_O_6_	*D*_x_ = 1.291 Mg m^−^^3^
*M**_r_* = 348.38	Mo *K*α radiation, λ = 0.71073 Å
Tetragonal, *P*4_3_2_1_2	Cell parameters from 6310 reflections
Hall symbol: P 4nw 2abw	θ = 2.5–32.6°
*a* = 13.014 (2) Å	µ = 0.10 mm^−^^1^
*c* = 21.167 (3) Å	*T* = 100 K
*V* = 3584.9 (9) Å^3^	Prism, colorless
*Z* = 8	0.37 × 0.25 × 0.25 mm
*F*(000) = 1488	

### Data collection

**Table:**

Nonius KappaCCD diffractometer	6507 independent reflections
Radiation source: sealed tube	5934 reflections with *I* > 2σ(*I*)
Horizonally mounted graphite crystal monochromator	*R*_int_ = 0.021
Detector resolution: 9 pixels mm^-1^	θ_max_ = 32.6°, θ_min_ = 3.1°
φ and ω scans	*h* = −19→19
Absorption correction: multi-scan (*SCALEPACK*; Otwinowski & Minor, 1997)	*k* = −13→13
*T*_min_ = 0.966, *T*_max_ = 0.977	*l* = −30→31
11378 measured reflections	

### Refinement

**Table:**

Refinement on *F*^2^	Secondary atom site location: difference Fourier map
Least-squares matrix: full	Hydrogen site location: inferred from neighbouring sites
*R*[*F*^2^ > 2σ(*F*^2^)] = 0.036	H-atom parameters constrained
*wR*(*F*^2^) = 0.092	*w* = 1/[σ^2^(*F*_o_^2^) + (0.0489*P*)^2^ + 0.548*P*] where *P* = (*F*_o_^2^ + 2*F*_c_^2^)/3
*S* = 1.02	(Δ/σ)_max_ = 0.001
6507 reflections	Δρ_max_ = 0.28 e Å^−^^3^
231 parameters	Δρ_min_ = −0.21 e Å^−^^3^
0 restraints	Absolute structure: Flack (1983). 2776 Bijvoet pairs
0 constraints	Flack parameter: 0.4 (6)
Primary atom site location: structure-invariant direct methods	

### Special details

**Table:**

Geometry. All e.s.d.'s (except the e.s.d. in the dihedral angle between two l.s. planes) are estimated using the full covariance matrix. The cell e.s.d.'s are taken into account individually in the estimation of e.s.d.'s in distances, angles and torsion angles; correlations between e.s.d.'s in cell parameters are only used when they are defined by crystal symmetry. An approximate (isotropic) treatment of cell e.s.d.'s is used for estimating e.s.d.'s involving l.s. planes.

### Fractional atomic coordinates and isotropic or equivalent isotropic displacement parameters (Å^2^)

**Table:**

	*x*	*y*	*z*	*U*_iso_*/*U*_eq_	
C1	0.99387 (7)	0.15194 (7)	0.38460 (4)	0.01487 (16)	
C2	1.10097 (8)	0.17487 (8)	0.41219 (5)	0.01852 (17)	
H2A	1.1216	0.2454	0.4001	0.022*	
H2B	1.0972	0.172	0.4589	0.022*	
C3	1.18311 (8)	0.09896 (9)	0.38923 (5)	0.02218 (19)	
H3A	1.2483	0.1131	0.4117	0.027*	
H3B	1.1952	0.11	0.3436	0.027*	
C4	1.15268 (8)	−0.01396 (8)	0.40011 (5)	0.02003 (18)	
H4	1.1451	−0.0238	0.4467	0.024*	
C5	1.04822 (8)	−0.03292 (8)	0.37083 (4)	0.01737 (17)	
C6	0.96687 (7)	0.03709 (7)	0.39832 (4)	0.01477 (15)	
H6	0.9717	0.0288	0.4452	0.018*	
C7	0.85545 (7)	0.01232 (8)	0.38106 (4)	0.01717 (17)	
H7A	0.8433	−0.0624	0.3855	0.021*	
H7B	0.8425	0.0315	0.3365	0.021*	
C8	0.78177 (8)	0.07130 (7)	0.42426 (4)	0.01629 (17)	
H8	0.711	0.0714	0.4058	0.02*	
C9	0.81884 (7)	0.17881 (7)	0.43274 (4)	0.01617 (16)	
C10	0.75719 (8)	0.26445 (8)	0.45837 (5)	0.01949 (18)	
C11	0.91383 (8)	0.32891 (8)	0.42864 (5)	0.01760 (17)	
H11	0.9261	0.3685	0.3889	0.021*	
C12	0.91114 (7)	0.21474 (7)	0.41591 (4)	0.01502 (16)	
C13	0.99210 (8)	0.17746 (8)	0.31324 (4)	0.02007 (18)	
H13A	0.9224	0.1675	0.2967	0.03*	
H13B	1.0398	0.1319	0.2908	0.03*	
H13C	1.013	0.2491	0.3069	0.03*	
C14	1.23690 (9)	−0.08734 (10)	0.37789 (6)	0.0301 (2)	
H14A	1.216	−0.1583	0.3865	0.045*	
H14B	1.3008	−0.0722	0.4005	0.045*	
H14C	1.2478	−0.0785	0.3324	0.045*	
C15	1.02974 (9)	−0.10108 (9)	0.32528 (5)	0.0227 (2)	
H15A	0.9625	−0.1076	0.3083	0.027*	
H15B	1.0838	−0.1431	0.3098	0.027*	
C16	1.02993 (9)	0.44956 (8)	0.47234 (5)	0.02094 (19)	
C17	1.10061 (10)	0.46776 (9)	0.52658 (5)	0.0266 (2)	
H17A	1.139	0.5315	0.5196	0.04*	
H17B	1.1487	0.4101	0.5302	0.04*	
H17C	1.0605	0.4736	0.5656	0.04*	
C18	0.71756 (8)	−0.05724 (8)	0.49413 (5)	0.02047 (18)	
C19	0.72455 (9)	−0.10197 (10)	0.55937 (5)	0.0262 (2)	
H19A	0.6552	−0.1138	0.5759	0.039*	
H19B	0.7611	−0.054	0.5871	0.039*	
H19C	0.7619	−0.1673	0.5577	0.039*	
O1	1.00912 (8)	0.50921 (7)	0.43108 (4)	0.0327 (2)	
O2	0.98905 (6)	0.35231 (6)	0.47497 (3)	0.01904 (14)	
O3	0.81477 (6)	0.35250 (6)	0.45508 (4)	0.02120 (15)	
O4	0.67051 (6)	0.26478 (7)	0.47800 (4)	0.02641 (17)	
O5	0.78024 (6)	0.02495 (6)	0.48730 (3)	0.01794 (14)	
O6	0.66441 (8)	−0.09022 (8)	0.45248 (4)	0.0323 (2)	

### Atomic displacement parameters (Å^2^)

**Table:**

	*U*^11^	*U*^22^	*U*^33^	*U*^12^	*U*^13^	*U*^23^
C1	0.0147 (4)	0.0157 (4)	0.0142 (4)	−0.0010 (3)	−0.0002 (3)	0.0010 (3)
C2	0.0154 (4)	0.0190 (4)	0.0212 (4)	−0.0020 (3)	−0.0014 (3)	−0.0006 (4)
C3	0.0148 (4)	0.0253 (5)	0.0265 (5)	0.0002 (4)	0.0006 (4)	−0.0006 (4)
C4	0.0184 (4)	0.0222 (5)	0.0195 (4)	0.0046 (3)	0.0007 (3)	−0.0013 (3)
C5	0.0191 (4)	0.0171 (4)	0.0159 (4)	0.0011 (3)	0.0017 (3)	0.0018 (3)
C6	0.0154 (4)	0.0153 (4)	0.0137 (4)	−0.0006 (3)	0.0001 (3)	0.0005 (3)
C7	0.0178 (4)	0.0176 (4)	0.0161 (4)	−0.0031 (3)	−0.0002 (3)	−0.0014 (3)
C8	0.0149 (4)	0.0186 (4)	0.0154 (4)	−0.0019 (3)	−0.0008 (3)	0.0012 (3)
C9	0.0158 (4)	0.0168 (4)	0.0160 (4)	0.0010 (3)	−0.0007 (3)	−0.0002 (3)
C10	0.0187 (4)	0.0206 (4)	0.0192 (4)	0.0020 (3)	−0.0018 (3)	−0.0011 (3)
C11	0.0185 (4)	0.0165 (4)	0.0178 (4)	0.0004 (3)	−0.0019 (3)	0.0008 (3)
C12	0.0162 (4)	0.0152 (4)	0.0137 (4)	0.0005 (3)	−0.0020 (3)	0.0008 (3)
C13	0.0241 (5)	0.0214 (4)	0.0147 (4)	−0.0002 (4)	0.0017 (3)	0.0038 (3)
C14	0.0222 (5)	0.0337 (6)	0.0342 (6)	0.0087 (4)	0.0005 (4)	−0.0060 (5)
C15	0.0255 (5)	0.0218 (5)	0.0208 (4)	0.0002 (4)	0.0027 (4)	−0.0028 (4)
C16	0.0249 (5)	0.0159 (4)	0.0220 (4)	−0.0026 (4)	0.0036 (4)	−0.0028 (3)
C17	0.0311 (6)	0.0235 (5)	0.0253 (5)	−0.0089 (4)	−0.0024 (4)	−0.0033 (4)
C18	0.0191 (4)	0.0222 (4)	0.0202 (4)	−0.0040 (4)	0.0008 (4)	0.0028 (4)
C19	0.0248 (5)	0.0304 (6)	0.0233 (5)	−0.0026 (4)	−0.0003 (4)	0.0089 (4)
O1	0.0492 (6)	0.0191 (4)	0.0299 (4)	−0.0068 (4)	−0.0052 (4)	0.0050 (3)
O2	0.0226 (4)	0.0154 (3)	0.0191 (3)	−0.0040 (3)	−0.0034 (3)	0.0015 (2)
O3	0.0197 (3)	0.0178 (3)	0.0261 (3)	0.0027 (3)	0.0010 (3)	−0.0020 (3)
O4	0.0181 (3)	0.0295 (4)	0.0316 (4)	0.0029 (3)	0.0022 (3)	−0.0046 (3)
O5	0.0172 (3)	0.0207 (3)	0.0159 (3)	−0.0035 (3)	−0.0009 (2)	0.0027 (3)
O6	0.0364 (5)	0.0361 (5)	0.0243 (4)	−0.0189 (4)	−0.0047 (4)	0.0026 (3)

### Geometric parameters (Å, º)

**Table:**

C1—C12	1.5054 (14)	C10—O3	1.3709 (13)
C1—C2	1.5403 (14)	C11—O2	1.4187 (12)
C1—C13	1.5468 (13)	C11—O3	1.4386 (13)
C1—C6	1.5627 (13)	C11—C12	1.5104 (14)
C2—C3	1.5346 (15)	C11—H11	1
C2—H2A	0.99	C13—H13A	0.98
C2—H2B	0.99	C13—H13B	0.98
C3—C4	1.5393 (16)	C13—H13C	0.98
C3—H3A	0.99	C14—H14A	0.98
C3—H3B	0.99	C14—H14B	0.98
C4—C5	1.5143 (14)	C14—H14C	0.98
C4—C14	1.5279 (15)	C15—H15A	0.95
C4—H4	1	C15—H15B	0.95
C5—C15	1.3321 (14)	C16—O1	1.1994 (14)
C5—C6	1.5131 (13)	C16—O2	1.3740 (12)
C6—C7	1.5297 (14)	C16—C17	1.4902 (15)
C6—H6	1	C17—H17A	0.98
C7—C8	1.5313 (14)	C17—H17B	0.98
C7—H7A	0.99	C17—H17C	0.98
C7—H7B	0.99	C18—O6	1.2000 (13)
C8—O5	1.4646 (12)	C18—O5	1.3529 (12)
C8—C9	1.4908 (14)	C18—C19	1.5013 (15)
C8—H8	1	C19—H19A	0.98
C9—C12	1.3373 (14)	C19—H19B	0.98
C9—C10	1.4766 (14)	C19—H19C	0.98
C10—O4	1.2021 (13)		
			
C12—C1—C2	112.03 (8)	O4—C10—O3	121.84 (10)
C12—C1—C13	107.62 (8)	O4—C10—C9	129.76 (10)
C2—C1—C13	110.01 (8)	O3—C10—C9	108.37 (8)
C12—C1—C6	106.06 (7)	O2—C11—O3	107.67 (8)
C2—C1—C6	108.56 (8)	O2—C11—C12	110.52 (8)
C13—C1—C6	112.55 (8)	O3—C11—C12	104.98 (8)
C3—C2—C1	112.68 (8)	O2—C11—H11	111.1
C3—C2—H2A	109.1	O3—C11—H11	111.1
C1—C2—H2A	109.1	C12—C11—H11	111.1
C3—C2—H2B	109.1	C9—C12—C1	124.74 (9)
C1—C2—H2B	109.1	C9—C12—C11	108.49 (9)
H2A—C2—H2B	107.8	C1—C12—C11	126.58 (9)
C2—C3—C4	112.83 (8)	C1—C13—H13A	109.5
C2—C3—H3A	109	C1—C13—H13B	109.5
C4—C3—H3A	109	H13A—C13—H13B	109.5
C2—C3—H3B	109	C1—C13—H13C	109.5
C4—C3—H3B	109	H13A—C13—H13C	109.5
H3A—C3—H3B	107.8	H13B—C13—H13C	109.5
C5—C4—C14	114.59 (9)	C4—C14—H14A	109.5
C5—C4—C3	108.98 (8)	C4—C14—H14B	109.5
C14—C4—C3	111.47 (9)	H14A—C14—H14B	109.5
C5—C4—H4	107.1	C4—C14—H14C	109.5
C14—C4—H4	107.1	H14A—C14—H14C	109.5
C3—C4—H4	107.1	H14B—C14—H14C	109.5
C15—C5—C6	123.58 (9)	C5—C15—H15A	120
C15—C5—C4	124.53 (9)	C5—C15—H15B	120
C6—C5—C4	111.88 (8)	H15A—C15—H15B	120
C5—C6—C7	116.39 (8)	O1—C16—O2	122.57 (10)
C5—C6—C1	110.31 (8)	O1—C16—C17	126.69 (10)
C7—C6—C1	111.73 (8)	O2—C16—C17	110.74 (9)
C5—C6—H6	105.9	C16—C17—H17A	109.5
C7—C6—H6	105.9	C16—C17—H17B	109.5
C1—C6—H6	105.9	H17A—C17—H17B	109.5
C6—C7—C8	110.20 (8)	C16—C17—H17C	109.5
C6—C7—H7A	109.6	H17A—C17—H17C	109.5
C8—C7—H7A	109.6	H17B—C17—H17C	109.5
C6—C7—H7B	109.6	O6—C18—O5	123.49 (10)
C8—C7—H7B	109.6	O6—C18—C19	124.89 (10)
H7A—C7—H7B	108.1	O5—C18—C19	111.61 (9)
O5—C8—C9	106.34 (7)	C18—C19—H19A	109.5
O5—C8—C7	110.25 (8)	C18—C19—H19B	109.5
C9—C8—C7	109.86 (8)	H19A—C19—H19B	109.5
O5—C8—H8	110.1	C18—C19—H19C	109.5
C9—C8—H8	110.1	H19A—C19—H19C	109.5
C7—C8—H8	110.1	H19B—C19—H19C	109.5
C12—C9—C10	108.79 (9)	C16—O2—C11	115.91 (8)
C12—C9—C8	125.92 (9)	C10—O3—C11	109.35 (8)
C10—C9—C8	125.23 (9)	C18—O5—C8	115.54 (8)
			
C12—C1—C2—C3	170.06 (8)	C8—C9—C10—O4	1.98 (17)
C13—C1—C2—C3	−70.28 (11)	C12—C9—C10—O3	1.20 (11)
C6—C1—C2—C3	53.28 (10)	C8—C9—C10—O3	−176.04 (9)
C1—C2—C3—C4	−53.11 (12)	C10—C9—C12—C1	−176.62 (8)
C2—C3—C4—C5	53.61 (11)	C8—C9—C12—C1	0.59 (15)
C2—C3—C4—C14	−178.93 (9)	C10—C9—C12—C11	−1.26 (10)
C14—C4—C5—C15	−5.67 (16)	C8—C9—C12—C11	175.95 (9)
C3—C4—C5—C15	120.00 (11)	C2—C1—C12—C9	−137.96 (10)
C14—C4—C5—C6	175.82 (9)	C13—C1—C12—C9	100.99 (11)
C3—C4—C5—C6	−58.51 (10)	C6—C1—C12—C9	−19.68 (12)
C15—C5—C6—C7	11.94 (14)	C2—C1—C12—C11	47.52 (12)
C4—C5—C6—C7	−169.54 (8)	C13—C1—C12—C11	−73.53 (11)
C15—C5—C6—C1	−116.70 (11)	C6—C1—C12—C11	165.80 (8)
C4—C5—C6—C1	61.83 (10)	O2—C11—C12—C9	116.73 (9)
C12—C1—C6—C5	−177.79 (7)	O3—C11—C12—C9	0.90 (10)
C2—C1—C6—C5	−57.24 (9)	O2—C11—C12—C1	−68.02 (12)
C13—C1—C6—C5	64.78 (10)	O3—C11—C12—C1	176.15 (8)
C12—C1—C6—C7	51.08 (9)	O1—C16—O2—C11	−4.93 (15)
C2—C1—C6—C7	171.64 (8)	C17—C16—O2—C11	174.94 (9)
C13—C1—C6—C7	−66.34 (10)	O3—C11—O2—C16	−92.25 (10)
C5—C6—C7—C8	166.12 (8)	C12—C11—O2—C16	153.62 (8)
C1—C6—C7—C8	−65.93 (10)	O4—C10—O3—C11	−178.80 (10)
C6—C7—C8—O5	−74.35 (10)	C9—C10—O3—C11	−0.60 (11)
C6—C7—C8—C9	42.52 (10)	O2—C11—O3—C10	−117.91 (9)
O5—C8—C9—C12	107.52 (10)	C12—C11—O3—C10	−0.14 (10)
C7—C8—C9—C12	−11.78 (13)	O6—C18—O5—C8	−1.47 (15)
O5—C8—C9—C10	−75.71 (11)	C19—C18—O5—C8	177.62 (9)
C7—C8—C9—C10	164.99 (9)	C9—C8—O5—C18	158.03 (8)
C12—C9—C10—O4	179.22 (11)	C7—C8—O5—C18	−82.93 (10)
